# Long-term Outcomes of Adjunctive Lung Resection for Nontuberculous Mycobacteria Pulmonary Disease

**DOI:** 10.1093/ofid/ofae345

**Published:** 2024-06-24

**Authors:** Noeul Kang, Byung Woo Jhun

**Affiliations:** Division of Allergy, Department of Medicine, Samsung Medical Center, Sungkyunkwan University School of Medicine, Seoul, South Korea; Division of Pulmonary and Critical Care Medicine, Department of Medicine, Samsung Medical Center, Sungkyunkwan University School of Medicine, Seoul, South Korea

**Keywords:** *Mycobacterium abscessus*, *Mycobacterium avium* complex, nontuberculous mycobacteria, outcome, surgery

## Abstract

**Background:**

Adjunctive lung resection is recommended for select patients with nontuberculous mycobacteria (NTM) pulmonary disease (PD). However, data are limited on long-term recurrence rates in patients infected with major pathogens, including *Mycobacterium avium* complex (MAC) and *Mycobacterium abscessus* (MABC).

**Methods:**

In this prospective observational study, we retrospectively analyzed data from 125 patients with MAC-PD (n = 90) or MABC-PD (n = 35) who underwent adjunctive lung resection. We evaluated microbiological response, postoperative complications, recurrence, and all-cause mortality over a median 80-month follow-up.

**Results:**

Persistent culture positivity (64%) was the most common indication for surgery, followed by hemoptysis, recurrent pneumonia, or radiologic deterioration. Postoperative complications occurred in 18 (14%) patients, with no surgery-related deaths. Treatment outcomes did not significantly differ between the MAC- and MABC-PD groups. Cure with culture conversion was achieved in 112 (90%) patients. Recurrence occurred in 37 (33%) of 112 patients, of which 18 (49%) cases were attributed to reinfection by different NTM species or subspecies. The MAC group had higher recurrence rates than the MABC group (Kaplan-Meier curve, log-rank test, *P* = .043) and was significantly associated with recurrence in the multivariable analysis (adjusted hazard ratio, 2.71; 95% CI, 1.23–5.99). However, mortality was higher in the MABC-PD group than the MAC-PD group (7/35 vs 4/90, *P* = .006).

**Conclusions:**

Adjunctive lung resection with antibiotics helps to reduce bacterial burden and manage symptoms in patients with NTM-PD. However, it does not prevent recurrence, which is mostly caused by reinfection.

## Teaser

Lung resection in addition to antibiotic therapy can reduce nontuberculous mycobacteria burden, facilitate culture conversion, and contribute to symptom control in patients with nontuberculous mycobacteria pulmonary disease. However, it does not prevent recurrence after microbiological cure, in which many cases are due to reinfection.

Nontuberculous mycobacteria (NTM) pulmonary disease (PD) is a chronic pulmonary infection caused by various NTM species, and the incidence and prevalence are rapidly increasing worldwide [1, 2]. The *Mycobacterium avium* complex (MAC), primarily composed of *M avium* and *M intracellulare*, is the most common causative agent of NTM-PD worldwide. The *Mycobacterium abscessus* (MABC), primarily composed of subspecies *abscessus* and subspecies *massiliense*, is a well-known treatment-refractory organism that is highly resistant to most antibiotics [3].

Treatment guidelines recommend maintaining a macrolide-based multidrug antibiotic therapy for at least 12 months after achieving culture conversion [3, 4]. For MAC infection, rifampicin and ethambutol are used as companion drugs, whereas the treatment of MABC infection involves the use of broad-spectrum antibiotics such as amikacin, imipenem, and tigecycline. However, most recommended antibiotics lack correlation between in vitro activity and clinical outcomes, and orally administered drugs with minimal side effects are rare. Consequently, long-term treatment outcomes are suboptimal. Culture conversion is achieved in only 60% to 70% and 50% of patients with MAC and MABC infections, respectively [5–7]. Thus, clinicians in real-world settings are constantly contemplating the use of adjunctive treatment methods.

A recent guideline recommends adjuvant lung surgery in selected patients with unsuccessful medical therapy, cavitary lesions, or complications such as severe bronchiectasis or hemoptysis [3]. According to a recent meta-analysis, lung surgery has the potential to reduce NTM loads [8]. However, surgery-related complications and mortality have been reported. Moreover, NTM-PD is inherently a multifactorial disease owing to the ubiquitous characteristics of NTM pathogens. Therefore, achieving culture conversion after surgery does not eliminate the possibility of recurrence during long-term follow-up. Yet, studies analyzing not only MAC but also MABC are limited, and studies evaluating long-term recurrence are scarce. Therefore, in this study, we investigated the outcomes of NTM-PD caused by 2 important etiologic agents, MAC and MABC, in patients who underwent adjuvant lung resection. We evaluated surgery-related complications and recurrence rates during long-term follow-up according to the etiologic agent. We hope that our data will assist in determining the need for lung surgery in patients with NTM-PD and in establishing follow-up strategies.

## METHODS

### Study Population

**Figure 1.**
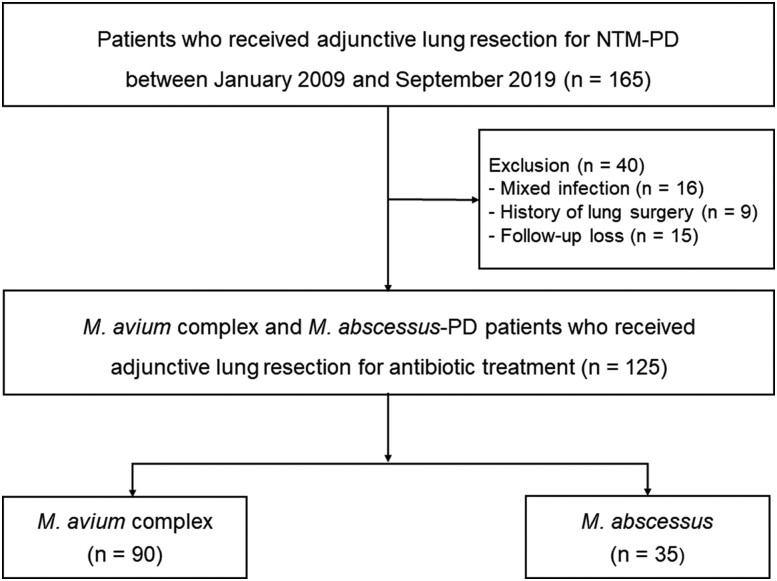
Study participants. *M abscessus*, *Mycobacterium abscessus*; *M avium*, *Mycobacterium avium*; NTM, nontuberculous mycobacteria; PD, pulmonary disease.

We screened 165 patients with MAC- or MABC-PD who underwent adjunctive lung resection in addition to antibiotic therapy between January 2009 and September 2019 from the NTM Registry of Samsung Medical Center, Seoul, South Korea. Data were obtained from an ongoing institutional review board–approved prospective observational cohort that began in 2008 (ClinicalTrials.gov: NCT00970801). We excluded 40 patients: those with mixed infections (n = 16) or a history of lung surgery (n = 9) and those who were lost to follow-up or transferred to another institution (n = 15). Finally, 125 patients with MAC (n = 90) or MABC-PD (n = 35) who had undergone adjunctive lung resection surgery were included in the analysis (Figure 1). All patients met the diagnostic criteria per the guidelines of the American Thoracic Society and Infectious Diseases Society of America [3, 9]. The follow-up data for the patients were last retrieved on 31 December 2023. Written informed consent was obtained from the participants, and the institutional review board of Samsung Medical Center approved the retrospective analysis and publication of prospectively collected data (No. 2024-03-058).

### Antibiotic Regimens and Indications for Lung Resection

The antibiotic regimens were based on recent guidelines [3, 9]. All patients with MAC-PD received macrolide-based regimens, including ethambutol and rifamycin, with or without intravenous aminoglycosides (amikacin or streptomycin) [10]. In patients with MABC-PD, multiple intravenous antibiotics, such as amikacin, imipenem, and tigecycline, were used during the initial phase for several months, and nonliposomal amikacin inhalation and oral agents, such as macrolides, clofazimine, linezolid, and fluoroquinolone, were used during the maintenance period for several months [11–13].

During antibiotic treatment, surgical lung resection was performed per clinical decision in the following cases: (1) persistent acid-fast bacilli on smear and/or culture despite antibiotic therapy for ≥12 months; (2) massive hemoptysis or recurrent pneumonia due to cavities or severe bronchiectasis lesions; or (3) rapid radiologic deterioration. Based on consultations between the attending physician and thoracic surgeon, the extent of pulmonary resection for lung surgery was determined, including options such as wedge resection, segmentectomy, lobectomy, or pneumonectomy. The indications for surgery in this study differed from those of our institution’s past study [14]. In this study, we selected patients for surgery, not after 6 months of antibiotic treatment, but after >12 months if the causative NTM pathogen persisted. Additionally, cases showing rapid radiologic deterioration were selected and not just those with remaining cavities or bronchiectasis. “Rapid radiologic deterioration” was defined as cases where the size of the cavity increases or new bronchiolitis/consolidation occurs around bronchiectasis on radiograph or computed tomography within 3 months despite antibiotic treatment.

### Microbiological and Radiologic Evaluation

Sputum examinations were usually performed at 1, 3, and 6 months after antibiotic initiation, followed by examinations at 2- to 3-month intervals. Acid-fast bacilli smear and culture of sputum specimens were performed via standard methods. Specimens were cultured on 3% Ogawa solid medium (Shinyang) and in liquid broth medium in mycobacterial growth indicator tubes (Becton, Dickinson and Co). Polymerase chain reaction–restriction fragment length polymorphism analysis or reverse blot hybridization of the *rpoB* gene was used to identify NTM species. From June 2014, NTM species were identified by nested multiplex polymerase chain reaction and a reverse hybridization assay of the internal transcribed spacer region (AdvanSure Mycobacteria GenoBlot Assay; LG Life Sciences). All patients underwent chest computed tomography at the time of starting treatment or during the follow-up period to evaluate radiologic characteristics or response to treatment. The radiologic form of the disease was categorized as fibrocavitary or nodular bronchiectatic based on the dominant presence of fibrocavities or bilateral bronchiectasis, as described in previous studies [10].

Microbiological outcomes were based on the NTM-NET consensus statement [15]. “Culture conversion” was defined as at least 3 consecutive negative cultures of sputum collected at least 4 weeks apart after treatment. The time of conversion was defined as the date of the first negative culture. “Cure” was defined as multiple consecutive negative cultures of the causative species after culture conversion until the completion of treatment. “Relapse” was defined as at least 2 positive cultures of the same species or subspecies emerging after completing treatment. “Reinfection” was defined as at least 2 positive cultures of a different species or subspecies emerging after completing treatment. For MAC-PD, the distinction is based on species (*M avium* or *M intracellulare*), and for MABC-PD, it is based on subspecies (*abscessus* or *massiliense*). “Recurrence” included relapse and reinfection.

### Statistical Analysis

Data are presented as number (percentage) for categorical variables and median (IQR) for continuous variables. Categorical variables were compared with the Pearson chi-square test or Fisher exact test, and continuous variables were compared by Student *t* test. To estimate the recurrence of NTM-PD after surgical resection, the Kaplan-Meier method was used. Hazard ratios and 95% CIs for the risk of recurrence were calculated through the Cox proportional hazard model. A 2-sided *P* < .05 was considered statistically significant. All statistical analyses were performed with Stata (version 14.0; Stata Corporation).

## RESULTS

### Patient Characteristics

The baseline characteristics of the study population at the time of starting treatment are summarized in Table 1. Of the 125 patients in the study, 90 and 35 had MAC-PD and MABC-PD, respectively. Among the patients with MAC-PD, 47 were infected with *M avium* and 43 with *M intracellulare*, while among the patients with MABC-PD, 26 were infected with subspecies *abscessus* and 9 with subspecies *massiliense*. The median age was 53 years, 75% of patients were female, and more than half (53%) had a history of NTM-PD treatment. Approximately one-third exhibited nodular bronchiectasis on chest computed tomography. The median percentage predicted forced expiratory volume in 1 second on pulmonary function tests was 80%. However, no significant differences in clinical characteristics were observed between the MAC- and MABC-PD groups.

**Table 1.** Patient Characteristics According to NTM Etiology

Characteristics	Total (n = 125)	MAC (n = 90)	MABC (n = 35)	*P* Value
Age, y	53 (48–58)	53 (48–59)	52 (48–57)	.598
Sex, female	94 (75)	65 (72)	29 (83)	.216
Body mass index, kg/m^2^	20.9 (19.2–22.4)	21.0 (19.3–22.5)	20.9 (19.2–22.3)	.864
Never smoker	98 (78)	69 (77)	29 (83)	.450
Previous pulmonary tuberculosis	58 (46)	38 (42)	20 (57)	.133
Previous NTM treatment^a^	66 (53)	52 (58)	14 (40)	.074
COPD	11 (9)	9 (10)	2 (6)	.726
Chronic pulmonary aspergillosis	3 (2)	2 (2)	1 (3)	.999
Diabetes mellitus	5 (4)	3 (3)	2 (6)	.619
Malignancy	5 (4)	4 (4)	1 (3)	.999
Radiologic forms				.761
Nodular bronchiectatic disease	83 (66)	57 (63)	26 (74)	
Without cavity	58/83	41/57	17/26	
With cavity	25/83	16/57	9/26	
Fibrocavitary form	42 (34)	33 (37)	9 (26)	
ESR, mm/h^b^	24 (12–45)	22 (12–41)	31 (12–56)	.247
Sputum AFB smear positive^b^	76 (61)	51 (57)	25 (71)	.129
Preoperative spirometry, predicted %				
FVC	81 (73–94)	82 (73–95)	80 (72–89)	.384
FEV_1_	80 (66–91)	82 (66–93)	76 (63–87)	.309

Data are presented as No. (%) or median (IQR).

Abbreviations: AFB, acid-fast bacilli; COPD, chronic obstructive pulmonary disease; ESR, erythrocyte sedimentation rate; FEV_1_, forced expiratory volume in 1 second; FVC, functional vital capacity; MABC, *Mycobacterium abscessus*; MAC, *Mycobacterium avium complex*; NTM, nontuberculous mycobacteria.

^a^Previous NTM treatment is defined as patients who previously received antibiotic treatment for at least 6 months for the causative NTM agent.

^b^These items were measured at the time of lung resection surgery.

### Treatment Modalities

The treatment modalities are presented in Table 2. The most common reason for pulmonary resection was persistent culture positivity (64%), followed by hemoptysis or recurrent pneumonia and rapid radiologic deterioration. Among the types of surgery, lobectomy was the most common (73%), followed by segmentectomy. Pneumonectomy was performed in 4 patients. Surgical resection of bronchiectasis lesions and areas with cavities was performed in approximately 59% and 45% of patients, respectively. Granulomas were observed in the majority (91%) of cases. The median time from starting antibiotics to surgery was 14 months, and the overall treatment duration was a median 29 months. Except for the longer overall follow-up period in the MABC group, no significant differences were observed between the groups. Additionally, there were no significant differences between the antibiotics used before and after surgery (Supplementary Table 1).

**Table 2.** Treatment Modalities According to NTM Etiology

Treatment Modalities	Total (n = 125)	MAC (n = 90)	MABC (n = 35)	*P* Value
Indication for surgery^a^				.399
Persistent AFB smear and/or culture positivity	80 (64)	59 (66)	21 (60)	
Symptom control: hemoptysis or recurrent pneumonia	23 (18)	14 (15)	9 (26)	
Rapid radiologic deterioration	22 (18)	17 (19)	5 (14)	
Type of surgery				.880
Wedge resection	5 (4)	4 (4)	1 (3)	
Segmentectomy	15 (12)	12 (13)	3 (9)	
Lobectomy	91 (73)	65 (72)	26 (74)	
Lobectomy and segmentectomy	10 (8)	6 (7)	4 (11)	
Pneumonectomy	4 (3)	3 (3)	1 (3)	
Histopathologic findings^a^				
Bronchiectasis	74 (59)	49 (54)	25 (71)	.083
Cavity	56 (45)	43 (48)	13 (37)	.283
Granuloma	114 (91)	86 (96)	28 (80)	.011
Time from starting antibiotics to surgery, mo	14 (8–21)	13 (8–20)	15 (7–22)	.663
Total treatment duration, mo	29 (23–38)	28 (22–37)	29 (24–48)	.176
Overall follow-up period, mo	80 (63–120)	77 (62–108)	109 (65–135)	.013

Data are presented as No. (%) or median (IQR).

Abbreviations: AFB, acid-fast bacilli; MABC, *Mycobacterium abscessus*; MAC, *Mycobacterium avium* complex; NTM, nontuberculous mycobacteria.

^a^More than 1 surgical indication and/or histopathologic finding may be present in each patient.

### Treatment Outcomes

The treatment outcomes and surgical complications for all patients are presented in Table 3. Culture conversion and cure occurred in 112 (90%) patients. Specifically, 87% of patients with *M avium*, 91% with *M intracellulare*, 88% with subspecies *abscessus*, and 100% with subspecies *massiliense* achieved culture conversion. Among them, 29% (32/112) reached culture conversion with antibiotic therapy before surgery, as opposed to 71% (80/112) after surgery. The median time from initiation of antibiotics to culture conversion for all patients was 13 months, and negative cultures were confirmed within 6 months in 25% (29/112) of patients.

**Table 3.** Treatment Outcomes According to NTM Etiology

Outcomes	Total (n = 125)	MAC (n = 90)	MABC (n = 35)	*P* Value
Culture conversion	112 (90)	80 (89)	32 (91)	.999
Before surgery	32	23 (29)	9 (28)	
After surgery	80	57 (71)	23 (72)	
Within 6 mo^a^	29	19	10	.413
Time from antibiotics to culture conversion, mo	13 (5–21)	13 (6–20)	11 (1–21)	.663
Postsurgery complications	18 (14)	13 (14)	5 (14)	.999
Prolonged air leak	6	5	1	
Pleural effusion	4	2	2	
Pneumothorax	4	3	1	
Pneumonia	3	3	…	
Bronchopulmonary fistula	1	…	1	
Recurrence	37/112 (33)	28/80 (35)	9/32 (28)	.485
Relapse by same species/subspecies^b^	19	17	2	
Reinfection by different species/subspecies^c^	18	11	7	
All-cause mortality	11 (9)	4 (4)	7 (20)	.006
Time from starting antibiotics to death, mo	96 (67–164)	60 (44–82)	109 (76–165)	.039
Time from surgery to death, mo	75 (42–144)	39 (29–58)	92 (75–144)	<.010

Data are presented as No. (%) or median (IQR).

Abbreviations: MABC, *Mycobacterium abscessus*; MAC, *Mycobacterium avium* complex; NTM, nontuberculous mycobacteria.

^a^Patients who achieved culture conversion within 6 months after the starting the entire treatment.

^b^At least 2 positive cultures of the same species or subspecies emerging after completing treatment.

^c^At least 2 positive cultures of a different species or subspecies emerging after completing treatment. Detailed data regarding original causative agents and recurrence agents in 37 patients with recurrence of NTM pulmonary disease are presented in the Supplementary Table 2.

Postoperative complications occurred in 18 patients, with prolonged air leak (n = 6) being the most common, followed by pleural effusion or pneumothorax, each occurring in 4 patients. Three patients experienced postoperative pneumonia, and 1 developed bronchopulmonary fistula. However, no significant differences in surgical complications were observed between the MAC and MABC-PD groups.

**Figure 2.**
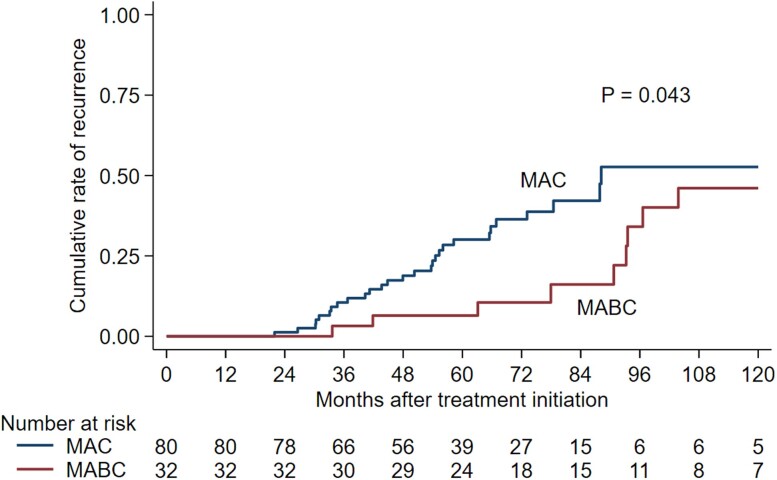
Cumulative recurrence rate in patients with nontuberculous mycobacteria pulmonary disease cured after antibiotic therapy and adjunctive surgery according to nontuberculous mycobacteria etiology. MABC, *Mycobacterium abscessus*; MAC, *Mycobacterium avium* complex.

Among the 112 patients who reached microbiological cure, recurrence occurred in 37 (33%). Among them, 51% (19/37) were infected with the same NTM species or subspecies as at the start of treatment, whereas the remaining 49% (18/37) were infected with different species or subspecies of NTM as compared with the start of treatment. Notably, among the 28 patients who experienced recurrence of NTM-PD after being cured of MAC-PD, 11 (39%) cases were due to different species or subspecies of NTM-PD. In contrast, among the 9 patients who experienced recurrence of NTM-PD after being cured of MABC-PD, 7 (78%) cases were due to different species. Detailed data regarding original causative agents and recurrence agents in 37 patients with recurrence of NTM-PD are presented in the Supplementary Table 2. Kaplan-Meier curves illustrating cumulative recurrence rates according to the MAC and MABC groups showed a higher cumulative recurrence rate in the MAC group (log-rank test, *P* = .043; Figure 2). However, throughout the study period, the mortality rate was higher in the MABC-PD group than in the MAC-PD group (20% vs 4%, *P* = .006). In a multivariate Cox proportional hazard analysis investigating factors related to all-cause mortality, no significant factors were found.

### Factors Associated With Postoperative Recurrence

Among the 112 patients who achieved microbiological cure, the clinical characteristics of the 37 who experienced recurrence and the 75 who did not are presented in Supplementary Tables 3 to 5. No significant differences were observed in the clinical characteristics of these patients, and 4 who underwent pneumonectomy were classified under the nonrecurrence group. We constructed models to analyze various factors related to recurrence using different variables, as shown in Table 4. The MAC species were significantly associated with recurrence (adjusted hazard ratio, 2.71; 95% CI, 1.23–5.99).

**Table 4.** Factors Associated With Recurrence After Treatment Completion With Achievement of Culture Conversion (n = 112)

Characteristics^a^	Adjusted Hazard Ratio (95% CI)	*P* Value
Age ≥65 y	0.91 (.19–4.22)	.900
Female	1.95 (.75–5.02)	.169
Body mass index ≤18.5 kg/m^2^	1.97 (.70–5.54)	.200
Previous pulmonary tuberculosis	0.80 (.38–1.68)	.556
Previous NTM treatment	0.49 (.24–1.03)	.058
MAC (vs MABC)	2.71 (1.23–5.99)	.014
Cavitary lesion (vs noncavitary)	0.77 (.37–1.57)	.466
Sputum AFB smear +	1.45 (.69–3.03)	.326
Time to culture conversion ≥6 mo	0.87 (.39–1.96)	.739

Abbreviations: AFB, acid-fast bacilli; MABC, *Mycobacterium abscessus* complex; MAC, *Mycobacterium avium* complex; NTM, nontuberculous mycobacteria.

^a^Clinical characteristics are indicators evaluated at the time of starting treatment for NTM pulmonary disease.

## DISCUSSION

This study summarizes the treatment outcomes and long-term recurrence rates in patients with MAC- or MABC-PD who underwent adjunctive lung resection in addition to antibiotic therapy. Our data support the effectiveness of lung resection in reducing the bacterial burden, facilitating sterilization, and aiding in controlling antibiotic-resistant respiratory disease in selected patients. Notably, culture conversion was achieved postoperatively in 80 patients for whom it did not occur despite antibiotic therapy, whereas 32 patients maintained their conversion status postoperatively. These findings suggest that adjunctive surgery can significantly improve treatment outcomes in cases resistant to medical therapy or patients experiencing persistent symptoms. Considering that augmented regimens for refractory MAC-PD, including liposomal amikacin inhalation or clofazimine, can result in an additional cure rate of approximately 30%, the achievement of culture conversion in 71% (57/80) of patients with MAC-PD after surgery in our study is noteworthy [16, 17]. Furthermore, given that the treatment response rate in MABC-PD is approximately 50% despite aggressive injectable antibiotics for several months, the 72% postoperative culture conversion rate (23/32) in this study represents a clinically significant finding [6, 13, 18, 19]. Therefore, our findings support the notion that adjunctive lung resection can enhance the clinical or microbiological response in select cases refractory to medical therapy. However, as emphasized in previous studies, meticulous patient selection and collaboration with well-trained surgeons are essential.

In this study, the recurrence rate was notably high at 33%. This contrasts with a recent meta-analysis of 841 patients from 15 studies, which included those who underwent adjunctive surgery for NTM-PD. Over a median follow-up of 34 months, the pooled recurrence rate was 9% [8]. Our higher rate may stem from our longer follow-up period, with a median 80 months from antibiotic treatment initiation. In a Japanese study of 114 patients with NTM-PD with a median postoperative follow-up of 69 months, 17% experienced recurrence, of whom 79% were infected with the same species [20]. In our study, recurrence was caused by the same species in 51% of patients and by a different species in the remaining 49%. Although genotyping was not conducted for the causative agents of recurrence in patients infected by the same species, our data suggest that reinfection plays a significant role in recurrence over time. Notably, in South Korean studies, the overall recurrence rate for MAC-PD is approximately 30% [21, 22]. Specifically, among 631 patients with MAC-PD who received medical treatment without lung surgery, the recurrence rate was approximately 32.4% [21]. These findings indicate that despite successful surgery, recurrence rates may be similar for those treated solely with long-term medical therapy. Thus, despite aggressive surgical interventions, reducing recurrence rates due to reinfection may be challenging. Given that NTM organisms are ubiquitous and NTM-PD occurs in patients with susceptible immune systems, this could be an expected outcome.

In this study, the Kaplan-Meier curve revealed that the cumulative recurrence rate tended to be higher in patients with MAC-PD over time, despite the shorter overall follow-up period in the MAC-PD group as compared with the MABC-PD group. Furthermore, multivariate analysis revealed that risk of recurrence was higher in the MAC group than in the MABC group. However, using this data set, we were unable to determine whether these phenomena occurred because patients infected with MAC had more vulnerable immune systems or there were other biases. Nevertheless, a notable finding from our data is that among the 28 cases that recurred after MAC clearance, only 39% (11/28) were reinfections by different species or subspecies, while the majority (78%, 7/9) that recurred after MABC clearance were reinfections caused by different species. These findings paradoxically suggest that adjunctive surgery may actively aid in eradicating the original species in patients with MABC-PD. Yet, this fact highlights the limitation that reinfection remains unavoidable even after MABC eradication through adjunctive lung resection. Recent studies have reported immune or metabolic alterations related to the pathogenesis of NTM-PD [23–25]. Nevertheless, no decisive measures to prevent reinfection have been reported. Further research on this topic is urgently required.

In this study, postoperative complications were observed in 14% of patients. This is slightly lower than the results of a recent meta-analysis, which reported that 17% of patients experienced postoperative complications [8]. This difference could be attributed to the relatively favorable preoperative pulmonary function or general conditions of our patients. In this study, prolonged air leak was the most common complication, and although postoperative pneumonia occurred, it was manageable. We believe that prolonged air leak and bronchopulmonary fistula have the maximum negative impact on patients’ quality of life after lung surgery. Owing to the risks associated with these complications, deciding on aggressive surgery is challenging in clinical practice, despite a high culture conversion rate with adjunctive surgery. Unfortunately, current treatment guidelines do not include well-defined criteria for selecting suitable candidates for adjunctive surgery, considering the risk-benefit profile. Therefore, further systematic data collection on this topic is required.

Our study had several limitations. Since the data are from a single center in an Asian country, generalizing the findings is difficult. Regarding recurrence, it was impossible to gather precise data on environments that may cause reinfection with NTM agents, potentially influencing the recurrence rate. Second, the skill level of the surgeon and the condition of the patient may have affected the microbiological cure and complication rates. Third, genotyping was not performed in our study. Reinfection was defined as an infection by a different species or subspecies and relapse as an infection by the same species or subspecies. This approach may underestimate the actual reinfection rate. In addition, there were cases of follow-up loss, which could have influenced the complications or mortality in this study. However, there were no surgery-related deaths or serious complications among these patients. Finally, the median duration from the initiation of antibiotic therapy to the decision for surgery was relatively long at 14 months, which could have influenced treatment outcomes. Unfortunately, current clinical guidelines on the timing of adjunctive lung resection surgery in patients who are refractory to medical therapy are unclear. Further data collection and analysis are required to address this issue.

## Supplementary Material

ofae345_Supplementary_Data

## Supplementary Data

Supplementary materials are available at *Open Forum Infectious Diseases* online. Consisting of data provided by the authors to benefit the reader, the posted materials are not copyedited and are the sole responsibility of the authors, so questions or comments should be addressed to the corresponding author.

## Notes

***Author contributions.*** Study conception and design, data acquisition and analysis, data interpretation, manuscript writing, and critical revision and final approval of the manuscript: N. K. and B. W. J.

***Availability of data and materials.*** Data not publicly available.

***Patient consent statement.*** Data were obtained from an ongoing institutional review board–approved prospective observational cohort, which began in 2008 (ClinicalTrials.gov: NCT00970801). Written informed consent was obtained from the participants, and the institutional review board of Samsung Medical collected data was approved for retrospective analysis and publication (No. 2024-03-058). This study complies with the Declaration of Helsinki and was performed according to ethics committee approval.
