# Supplementary material for: Long-term Outcomes of Adjunctive Lung Resection for Nontuberculous Mycobacteria Pulmonary Disease
Source: Open Forum Infect Dis. 2024 Jun 24;11(7):ofae345. doi: 10.1093/ofid/ofae345 (PMC11222975; doi:10.1093/ofid/ofae345)
Supplement: ofae345_Supplementary_Data [file ofae345_supplementary_data.docx]

Supplementary file

**Long-term outcomes of adjunctive lung resection for nontuberculous mycobacteria pulmonary disease**

Noeul Kang^1^, Byung Woo Jhun^2^

^1^Division of Allergy, Department of Medicine, Samsung Medical Center, Sungkyunkwan University School of Medicine, Seoul, South Korea

^2^Division of Pulmonary and Critical Care Medicine, Department of Medicine, Samsung Medical Center, Sungkyunkwan University School of Medicine, Seoul, South Korea

**Supplementary table 1.** List of antibiotics used before and after adjunctive lung resection surgery (n = 125)

| **Antibiotics** | **Before surgery** | **After surgery** |
| --- | --- | --- |
| Macrolide | 125 | 124 |
| Ethambutol | 86 | 86 |
| Rifampicin | 81 | 79 |
| Clofazimine | 18 | 25 |
| Inhaled non-liposomal amikacin | 16 | 16 |
| Intravenous amikacin | 2 | 1 |
| Fluoroquinolone | 15 | 10 |
| Streptomycin | 12 | 4 |
| Linezolid | 4 | 5 |
| Cefoxitin | 1 | - |

**Supplementary table 2.** Comparison of original causative agents and recurrence agents in 37 patients with recurrence of NTM-PD

| **Original causative agents** | **Agents causing recurrence** | **No. of patients** |
| --- | --- | --- |
| M. avium (n = 14) | M. avium | n = 6 |
|  | M. intracellulare | n = 5 |
|  | M. abscessus subspecies abscessus | n = 3 |
| M. intracellulare (n = 14) | M. intracellulare | n = 11 |
|  | M. avium | n = 3 |
| M. abscessus subspecies abscessus (n = 6) | M. abscessus subspecies abscessus | n = 2 |
|  | M. avium | n = 3 |
|  | M. intracellulare | n = 1 |
| M. abscessus subspecies massiliense (n = 3) | M. intracellulare | n = 2 |
|  | M. avium | n = 1 |

**Supplementary table 3.** Characteristics according to recurrence after treatment completion with achievement of culture conversion

| **Characteristics** | **Total**  **(n = 112)** | | **Non-recurrence**  **(n = 75)** | | **Recurrence**  **(n = 37)** | | **p-value** |
| --- | --- | --- | --- | --- | --- | --- | --- |
| Age, years | 53 (49 – 58) | 54 (45 – 59) | | 52 (50 – 57) | | 0.466 | |
| Sex, female | 29 (26) | 23 (31) | | 6 (16) | | 0.101 | |
| Body mass index, kg/m^2^ | 20.9 (19.2 – 22.3) | 21.0 (19.1 – 22.5) | | 20.9 (19.5 – 22.3) | | 0.567 | |
| Never smoker | 86 (77) | 53 (71) | | 33 (89) | | 0.029 | |
| Previous pulmonary tuberculosis | 52 (46) | 39 (52) | | 13 (35) | | 0.092 | |
| Previous NTM treatment | 59 (53) | 47 (63) | | 12 (32) | | 0.003 | |
| COPD | 10 (9) | 5 (7) | | 5 (14) | | 0.294 | |
| Chronic pulmonary aspergillosis | 2 (2) | 1 (1) | | 1 (3) | | 0.999 | |
| Diabetes mellitus | 5 (4) | 5 (7) | | - | | 0.169 | |
| Malignancy | 5 (5) | 4 (5) | | 1 (3) | | 0.999 | |
| *Mycobacterium* species |  |  | |  | | 0.320 | |
| MAC | 80 (71) | 52 (70) | | 28 (76) | |  | |
| MABC | 32 (29) | 23 (30) | | 9 (24) | |  | |
| Radiological forms |  |  | |  | | 0.139 | |
| Nodular bronchiectatic disease | 71 (63) | 44 (59) | | 27 (73) | |  | |
| Without cavity | 49/71 | 31/44 | | 18/27 | |  | |
| With cavity | 22/71 | 13/44 | | 9/27 | |  | |
| Fibrocavitary form | 41 (37) | 31 (41) | | 10 (27) | |  | |
| Sputum AFB smear positive | 66 (59) | 42 (56) | | 24 (65) | | 0.370 | |
| ESR, mm/h | 22 (11 – 48) | 17 (11 – 34) | | 32 (12 – 56) | | 0.038 | |
| Pre-operative spirometry |  |  | |  | |  | |
| FVC, pred% | 81 (72 – 95) | 80 (70 – 95) | | 82 (76 – 92) | | 0.863 | |
| FEV_1_, pred% | 81 (67 – 93) | 78 (65 – 92) | | 81 (70 – 93) | | 0.712 | |

Data are presented as number (percentage) or median (interquartile range).

AFB, acid-fast bacilli; COPD, chronic obstructive pulmonary disease; ESR, erythrocyte sedimentation rate; FEV_1_, forced expiratory volume in 1 second; FVC, functional vital capacity; MABC, *Mycobacterium abscessus* complex; MAC, *Mycobacterium avium* complex; NTM, nontuberculous mycobacteria.

**Supplementary table 4.** Treatment modalities according to recurrence after treatment completion with achievement of culture conversion

| **Treatment modalities** | **Total**  **(n = 112)** | **Non-recurrence**  **(n = 75)** | **Recurrence**  **(n = 37)** | **p-value** |
| --- | --- | --- | --- | --- |
| Indication for surgery |  |  |  | 0.480 |
| Persistent AFB smear and/or culture positivity | 69 (62) | 46 (61) | 23 (62) |  |
| Symptom control (hemoptysis or recurrent pneumonia) | 22 (19) | 13 (17) | 9 (24) |  |
| Rapid radiologic deterioration | 21(19) | 16 (22) | 5 (14) |  |
| Type of surgery |  |  |  | 0.018 |
| Wedge resection | 5 (4) | 2 (3) | 3 (8) |  |
| Segmentectomy | 14 (13) | 13 (17) | 1 (3) |  |
| Lobectomy | 81 (72) | 53 (71) | 28 (76) |  |
| Lobectomy and Segmentectomy | 8 (7) | 3 (4) | 5 (14) |  |
| Pneumonectomy | 4 (4) | 4 (5) | - |  |
| Histopathological findings |  |  |  |  |
| Bronchiectasis | 65 (58) | 41 (55) | 24 (65) | 0.304 |
| Cavity | 52 (46) | 34 (45) | 18 (49) | 0.741 |
| Granuloma | 103 (92) | 68 (91) | 35 (95) | 0.715 |
| Time from starting antibiotics to surgery, months | 13 (8 – 20) | 13 (8 – 19) | 14 (7 – 23) | 0.237 |
| Total treatment duration, months | 27 (22 – 35) | 26 (21 – 33) | 33 (24 – 38) | 0.104 |
| Overall follow-up period, months | 81 (61 – 126) | 69 (55 – 88) | 120 (99 – 151) | <0.001 |

Data are presented as number (percentage) or median (interquartile range).

AFB, acid-fast bacilli.

**Supplementary table 5.** Treatment outcomes according to recurrence after treatment completion with achievement of culture conversion

| **Outcomes** | **Total**  **(n = 112)** | **Non-recurrence**  **(n = 75)** | **Recurrence**  **(n = 37)** | **p-value** |
| --- | --- | --- | --- | --- |
| Culture conversion |  |  |  | 0.485 |
| Before surgery | 32 (29) | 23 (31) | 9 (24) |  |
| After surgery | 80 (71) | 52 (69) | 28 (76) |  |
| Culture conversion within 6 months | 29 (26) | 20 (27) | 9 (24) | 0.999 |
| Time from antibiotics to culture conversion, months | 13 (5 – 21) | 12 (5 – 19) | 17 (7 – 26) | 0.137 |
| Post surgery complications | 15 (13) | 10 (13) | 5 (14) | 0.999 |
| Prolonged air leak | 6/15 (40) | 5/10 (50) | 1/5 (20) |  |
| Pneumonia | 3/15 (20) | 2/10 (20) | 1/5 (20) |  |
| Pleural effusion | 2/15 (13) | - | 2/5 (40) |  |
| Pneumothorax | 3/15 (20) | 3/10 (30) | - |  |
| Bronchopulmonary fistula | 1/15 (7) | - | 1/5 (20) |  |
| All-cause mortality | 9 (8) | 7 (9) | 2 (5) | 0.472 |
| Time from starting antibiotics to death, months | 76 (67 – 98) | 76 (52 – 96) | 132 (98 – 165) | 0.180 |
| Time from surgery to death, months | 74 (42 – 92) | 69 (35 – 75) | 118 (92 – 144) | 0.149 |

Data are presented as number (percentage) or median (interquartile range).

**FIGURE LEGENDS**

**Figure 1.** Study participants

**Figure 2.** Cumulative recurrence rate in patients with NTM-PD cured after antibiotic therapy and adjunctive surgery according to NTM etiology.
